# Measuring thymic output across the human lifespan: a critical challenge in laboratory medicine

**DOI:** 10.1007/s11357-025-01555-3

**Published:** 2025-02-13

**Authors:** Vera Middelkamp, Eliisa Kekäläinen

**Affiliations:** 1 Translational Immunology Research Program and Dept. of Bacteriology and Immunology, University of Helsinki, Helsinki, Finland; 2 Center for Translational Immunology, University Medical Center Utrecht, Utrecht University, Utrecht, the Netherlands; 3 Clinical Microbiology, HUS Diagnostic Center, Helsinki University Hospital, Helsinki, Finland

**Keywords:** Recent thymic emigrants, Immunosenescence, T cells, T cell receptor extraction circle (TREC), Thymic involution, Thymic output, The thymus

## Abstract

Age-associated thymic involution leads to a significant decline in thymic T cell output, a major contributor to immunosenescence in the elderly. Accurately measuring thymic output is therefore critical for understanding the mechanisms behind immune aging. Furthermore, robust quantification of thymic output is essential in various other clinical and research settings, including the diagnosis of immunodeficiencies and the monitoring of T cell reconstitution following therapeutic interventions like hematopoietic stem cell transplantation. Current methodologies for measuring thymic output include T cell receptor excision circle (TREC) quantification via quantitative polymerase chain reaction and the enumeration of recent thymic emigrants (RTEs) using flow cytometry. However, TREC-based assays are inherently insensitive to subtle changes in thymic output, limiting their applicability beyond neonatal immunodeficiency screening. Similarly, RTE enumeration presents challenges; while surface markers exist for CD4⁺ RTEs, validated markers for CD8⁺ cytotoxic T lymphocytes are lacking. This represents a significant knowledge gap, particularly as aging has been shown to disproportionally affect the CD8 T cell pool. Moreover, while flow cytometry effectively measures mature naïve T cells, these cells do not accurately represent real-time thymic output, as they can persist in peripheral circulation for extended periods. These limitations highlight the pressing need for more accurate and sensitive methods to assess thymic output. Improved measurement techniques would not only enhance our understanding of thymic involution in the context of aging but also enable large-scale investigations into thymic function and the mechanisms driving its decline in both health and disease. In this review, we examine current methodologies for measuring thymic output in humans, critically evaluate their limitations, and discuss emerging approaches to address these gaps in the field.

## Introduction

With advancing age, the immune system progressively loses efficacy, leading to increased susceptibility to opportunistic infections, autoimmune diseases, and both the incidence and burden of cancer, in a process called immunosenescence [1]. This decline significantly compromises the immune system’s ability to recover from external insults, contributing to higher morbidity and mortality in the elderly [2]. A key driver of this immune degeneration is thymic involution, which results in diminished production of T cells—critical effector cells of the cellular immune system. However, the precise mechanisms and kinetic underlying thymic involution remain poorly understood [1, 3]. Recent studies have highlighted the clinical relevance of assessing thymic function, demonstrating that a genetic predisposition to lower thymic output is associated with an increased risk of severe COVID-19, independent of age. This finding emphasizes the importance of individual differences in thymic output in shaping susceptibility to common infections [4]. Despite its importance, genetic and environmental factors influencing the rate of thymic involution and thymic function in humans remain understudied, largely due to limitations in current methods for assessing thymic output. While diagnostic assays for thymic output, such as T cell receptor excision circle (TREC) quantification, are currently used in the diagnosis of inborn errors of immunity, their clinical application could be expanded to broader contexts if more robust and precise methodologies were developed. In this minireview, we critically evaluate existing techniques used for measuring thymic output, including TREC quantification and enumeration of recent thymic emigrants (RTEs) with flow cytometry. We argue that more precise methods to measure thymic output are urgently needed, particularly for applications such as predicting responses to cancer treatments.

**Fig. 1:**
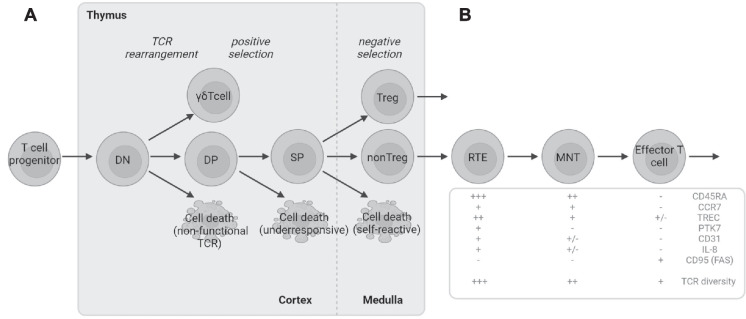
**A** Main developmental stages of T cell development in the thymus. Double negative (DN) cells lack both CD4 and CD8, while double positive (DP) cells express both lineage markers. Single-positive (SP) cells have committed to one or the other major T cell lineages: CD4-positive helper T cells or CD8-positive cytotoxic T cells. The thymus also produces several innate-like T cell populations, such as γδ-T-cells. Developing thymocytes undergo positive selection in the thymic cortex, where only those with functional T cell receptors (TCRs) that sufficiently recognize antigen-HLA-complexes can proceed to the thymic medulla. In the medulla, highly specialized medullary epithelial cells prune overly self-reactive thymocytes out of the repertoire before the T cell development is finalized and newly produced T cells can egress from the thymus to the periphery as recent thymic emigrants (RTE). The RTE population also contains thymic regulatory T cells that follows a distinct differentiation program in the medulla. **B** RTEs mature into mature naïve T cells (MNTs) after receiving additional signals in the peripheral immune system. MNTs are long-lived quiescent cells that can functionally mature into effector cells upon encountering their cognate antigen presented by antigen-presenting cell. Table 1 summarizes the key immunophenotypic features of each peripheral maturation stage

T cells undergo their primary developmental stages in the thymus, where they exit into the periphery as RTEs [5]. Studies using transgenic mouse models have demonstrated that RTEs represent a phenotypically and functionally distinct population. However, these antigen-inexperienced cells continue to mature extrathymically, rapidly differentiating into mature naive T cells (MNTs) [6–10]. MNTs are characterized by the expression of the CD45RA isoform, as well as receptors essential for lymph node homing, such as CCR7 and CD62L. In contrast to RTEs, MNTs are long-lived and, upon encountering their cognate antigen, can differentiate into effector and memory T cells (see Fig. 1) [6]. However, the longevity of MNTs makes them less suitable as a reliable measure of thymic output.

While studies in mice have provided valuable insights into the cellular and molecular mechanisms of thymic aging, such investigations are not feasible in humans. However, recent advances in single-cell transcriptomics have allowed for a detailed examination of circulating human naïve T cells. In the aging human immune system, not only is the proportion of MNTs reduced but their overall quantity is also affected [11, 12]. Moreover, humans rely primarily on homeostatic proliferation to maintain the naïve T cell pool [13], and age-associated thymic atrophy is far more pronounced in humans than in mice [14]. These findings underscore fundamental differences in T cell homeostasis between the two species over a lifetime.

The decline in thymic function begins early in human life. By early childhood, thymic tissue mass and size start to decrease significantly [15–17]. Notably, thymic epithelial cells, which form the microanatomical framework essential for T cell development, are particularly affected, with up to 70% of their population lost before the age of 20, and is replaced by adipose tissue [16, 17]. During adulthood, thymic involution continues at a slower rate, maintaining a low but relatively stable T cell output. However, by middle age, thymic function declines more abruptly and nearly ceases [13, 18, 19].

Despite this decline, thymic epithelial cells and signs of T cell development can still be detected even in very aged thymuses, suggesting that some capacity for T cell production remains, albeit at a diminished level [16, 20]. Recently, a large registry study by Kooshesh et al. proposed that thymectomy—the surgical removal of the thymus—in middle-aged or older adults is associated with increased overall mortality and a higher incidence of cancers [21]. However, we were unable to replicate this increase in mortality using Finnish registry data [22]. In certain autoimmune diseases, thymectomy improves prognosis and is therefore considered beneficial [23]. Partial or total thymectomy is also routinely performed during pediatric open-heart surgery, leading to temporary T cell lymphopenia; however, its long-term clinical consequences remain poorly understood [24]. Thus, thymic function and its role in immunosenescence remain relevant from childhood all the way to geriatric age. Nevertheless, many open questions remain, particularly regarding the differential effects of aging on CD4 + T cells and CD8 + T cells, as current evidence indicates aging and adverse metabolic factors may disproportionately reduce the naïve CD8 + T cell pool [25, 26].

Deepening our understanding of the biological mechanisms driving thymic involution, and the environmental factors influencing its progression, could facilitate the development of preventive measures to delay thymic ageing or regenerative approaches to rejuvenate thymic function [2]. Crucially however, unraveling the complexities of human thymic aging hinges on our ability to accurately measure thymic output, but current methodologies remain suboptimal, posing a significant barrier to fully decipher the impact of thymic involution on immunosenescence.

## Diagnostic and clinical applications of thymic output assessment

Measurement of thymic output serves as a valuable tool in clinical practice, particularly for diagnosing inborn errors of immunity. The absence or a very low proportion of naïve CD4 T cells is one of the diagnostic criteria for severe combined immunodeficiency (SCID), the most severe form of combined immunodeficiency [27]. For instance, national neonatal screening programs to detect SCID, based on the measurement of thymic output, have been successfully implemented in several countries [28, 29]. Approximately 30% of cases with abnormal newborn screening result will have a SCID diagnosis. Because the genetic causes of SCID are variable, in some rare cases, screening can miss certain atypical SCID types where the genetic pathology affects T cell function more than their development [27]. Additionally, reduced thymic output is often detected in other inborn errors of immunity defined by reduced or dysfunctional B and T cells [27, 30].

In adult patients that have been diagnosed with common-variable immunodeficiency (CVID), a condition mostly affecting B cells and antibody levels, the low proportion of naïve CD4 T cells is associated with more complications and a poor prognosis [31]. In CVID patients, the measurement of thymic output can help in evaluating if the patient’s diagnosis should be relabeled as a combined immunodeficiency, although the differentiation of patients with clinically relevant combined immunodeficiency from those with CVID remains a challenge [31]. Moreover, measuring thymic output allows for monitoring the reconstitution of the T cell pool after therapeutic interventions such as hematopoietic stem cell transplantation [32].

## T cell receptor excision circles (TRECs) are the current gold standard for measuring thymic output

Imaging methods, such as thoracic computed tomography (CT) and magnetic resonance imaging (MRI), have been used to determine thymic mass or volume. However, no clear correlation exists between thymus size and thymic output [33]. Therefore, it is only a semi-quantitative measurement and cannot reliably be used to assess thymic output. Using a ratio to measure the fat and soft tissue content of the thymus improves the accuracy of thymic imaging in assessing premature thymic involution; however, this method is not used in clinical practice [26]. Measuring thymic output in humans thus relies on laboratory tests, such as T cell receptor excision circles (TREC) assays [34]. During the T cell receptor (TCR) rearrangement in the thymus (Fig. 1), δ-coding segments between the TRAV and TRAJ genes are excised when the TCR α-genes are recombined [35]. The excised DNA is circularized due to the ligation of blunt DNA signal ends, forming circular excision products known as TRECs, which are stable extrachromosomal DNA fragments [35]. In clinical practice, TRECs are measured using quantitative polymerase chain reaction (qPCR). This assay is the cornerstone for newborn screening programs [28, 29], where the test is typically performed using dried whole blood spots collected on Guthrie cards, a convenient sample material that enables automation and high throughput processing [36]. TREC measurement can also be performed on isolated peripheral mononuclear cells, and this approach can be used, e.g., for monitoring after hematopoietic stem cell transplantation. In adults, the TREC assay should ideally be performed on purified T cells to enhance sensitivity [37], but this more complex procedure is available only in specialized laboratories (e.g. Mayo Clinic Laboratories and Great Ormond Street Laboratory in London) and not generally used in clinical diagnostics.

In the thymus, approximately 70% of immature thymocytes contain TRECs. However, during thymocyte maturation, TRECs are diluted up to 256-fold, with further dilution occurring in the periphery as TRECs are not replicated and therefore are diluted with each cell division [38]. TREC levels in CD4 + T cells correspond with thymic involution as a decline of 50–100 times is observed with aging [39]. Typically, TREC levels are above 10.000–20.000 copies per million cells in children, while the copy numbers are more variable in adults [40]. However, there are numerous approaches to TREC quantification, which makes a comparison between different studies almost impossible. TREC reconstitution is observed following hematopoietic stem cell transplantation in SCID patients [41] and after thymic tissue transplantation for complete congenital athymia [42]. In contrast, reduced TREC copies are commonly seen in individuals living with HIV [43, 44].

However, the method has several disadvantages that limits its usability beyond infancy. Firstly, since TREC content is influenced by the number of cell divisions, it is not a direct measure of thymic export [45]. TRECs can only be compared at the cell population level because, by definition, only one of the daughter cells of a TREC-positive cell retains the TREC. Similarly, not all RTEs are TREC positive due to intrathymic proliferation [38]. Secondly, due to the extremely low copy numbers, TREC assays cannot reliably be used in individuals over 70 years of age [46]. TREC levels are heavily influenced by the number and functional status of naïve T cells, making it difficult to compare TREC levels across individuals [37, 40, 45]. Substantial variability in the frequency of TREC positive cells has been observed, with greater variability in middle-aged individuals [40]. Thirdly, biological sex might be a factor influencing TREC copies. On average, women have 66–86% higher TREC values than men of all age ranges, reflecting the fact that age-related thymic involution occurs more prominently in males than in females [26, 47]. However, no difference in TREC copy numbers between sexes was detected in a large healthy cohort in Hong Kong [48]. Lastly, due to the nature of the assay, DNA extraction is required for TREC analysis, which destroys the cells and prevents further functional studies [34, 44]. Considering these limitations, TRECs alone cannot be used as a definitive measure of thymic output or immunological disease status, especially in adults.

## Lack of reliable cell surface markers for accurate thymic output evaluation via immunophenotyping

Lymphocytes can be isolated from blood samples and their maturation status detected with flow cytometry based immunophenotyping [49]. A reliable set of markers capable of comprehensively identifying cells as RTEs would enable a direct and accurate measure of thymic output. In humans, naïve T cells can be distinguished from other peripheral T cell populations through the expression of several cell surface proteins. For instance, CD45RA is expressed on naïve and stem cell memory cells, but not on central memory and effector memory cells, although it is re-expressed on terminal effector memory cells [49]. CCR7 is expressed by naïve T cells, stem cell memory, and central memory cells but not effector memory or terminal effector memory cells [50]. Therefore, combining these markers uniquely identifies naïve T cells within the general T cell population (see Table 1).

**Table 1:** Key T cell markers and their expression pattern throughout T cell development

	DN	DP	SP	Naïve T cells: RTEs	Naïve T cells: MNTs	Stem cell memory cells	Central memory cells	Effector memory cells	Terminal effector memory cells
CD45RA	+ / −	−	+	+	+	+	−	−	+
CD45RO	−	+	on 80%	−	−	−	+	+	-
CCR7	−	−	+	+	+	+	+	−	−
CD31	+	+	High on CD8s, low on CD4s until they are egress-ready	CD4s only	low on CD4s	−	−	−	−

+ = expressed, - = not expressed, +/- = variable expression

However, differentiating human naïve T cells into RTEs and MNTs has proven challenging. Despite phenotypic differences between RTEs and MNTs, no definitive cell surface markers have been identified, particularly for CD8 RTEs. For CD4 naïve T cells, CD31 (PECAM-1) is a widely accepted marker for RTEs [51, 52]. It correlates with TREC content, is rarely expressed on CD4 + memory cells, and shows declining expression with aging [28, 53]. The proportion of CD31 + naïve T cells has been shown to significantly contract in thymectomized individuals [54] and increase during immune reconstitution following allogeneic hematopoietic stem cell transplantation (HSCT) [32]. The low replicative history observed in CD31 + CD4 naïve T cells aligned with what would be expected of an RTE [52]. However, CD31 expression is maintained even in the elderly, strongly indicating that not all CD4 naïve T cells expressing CD31 represent RTEs [13]. This is further supported by evidence that IL-7-driven proliferation can occur in RTEs without downregulation of CD31 and that CD31 can be re-expressed on activated CD4 + T cells [55, 56]. Moreover, CD31 expression on CD8 naïve T cells does not correlate with TREC content, thymus size, or age, indicating it cannot be used as a marker for CD8 RTEs [57, 58]. Similarly, it fails as a marker for RTEs in ƴδ-T cells, an atypical innate-like T cell population also produced by the thymus [57].

Additionally, PTK7 has been suggested as an RTE marker in CD4 + naïve T cells. Its expression correlates with TRECs, CD31 expression, and age, and it declines following thymectomy [59, 60]. PTK7 + naïve CD4 T cells have undergone fewer cell divisions in the periphery compared to CD31 + cells. Furthermore, most PTK7 + naïve CD4 + T cells are also CD31 + , suggesting that PTK7 + cells represent a subpopulation of CD31 + CD4 + T cells and may constitute only the RTEs that have undergone the fewest cell divisions [59]. Thus, PTK7 has been used occasionally as a marker of CD4 + thymic export in research settings, typically in combination with CD31 [61]. However, its reliability as a RTE marker has also been questioned [62]. Commercial PTK7 antibodies for humans have proven to be unreliable, with unsatisfactory separation between PTK7-positive and negative cells, limiting their clinical utility [63]. The role and use of PTK7 as a marker for CD4 + RTEs remain uncertain.

Other markers have been proposed for CD8 RTEs, such as CD103, CD21, CD35, and IL-8 production [64–66]. However, none of these have been sufficiently validated in large cohorts to serve as reliable markers for CD8 RTEs. Recently, CD38 was suggested as a reliable CD8 RTE marker, although its clinical applicability remains to be shown [67].

## Improved methods of measuring thymic output could open new applications for both clinical and basic researches

Improved methods and assays for measuring thymic output could advance our understanding of thymic involution and its role in immunosenescence. Importantly, our current knowledge of the factors contributing to premature thymic decline is limited. By directly and accurately measuring thymic output, we could deepen our understanding of thymic function and T cell biology, in both health and disease. For example, since thymic involution progresses with age and RTEs are more abundant in younger individuals, improved measurement of thymic output could help clarify the characteristics of the neonatal adaptive immune system. This understanding is essential for optimizing the design of immune-based interventions, such as vaccines, in early life [68]. Additionally, it could enhance our knowledge of the mechanisms underlying homeostatic proliferation of naïve T cells in humans and its implications for health and disease [69].

Improved measurement of thymic output, or more precise identification of RTEs, could also expand our understanding of how individual differences in T cell production impact the outcome of infections [4]. On the other hand, infections can cause acute thymic atrophy, for which the long term impact on thymic output is unknown [70]. Similarly, states of low-grade inflammation, such as in obesity, have been linked to the degree of thymic involution [26]. More research is needed to fully understand the causes and long-term clinical consequences of normal and premature thymic involution, including its role in cancer development or autoimmune diseases. This can only be studied effectively by the accurate measurement of thymic output, both qualitatively and quantitatively, in large prospective patient cohorts.

Advanced methods for measuring thymic output would improve disease monitoring, especially in conditions that rely on the evaluation of thymic output and could also help predict clinical outcomes. For instance, monitoring of thymic output before and during immune reconstitution following HSCT has significantly improved our understanding of how the thymus is involved in the main complications related to HSCT [71, 72]. High TREC levels after HSCT in leukemia patients have been associated with a reduced relapse incidence, as well as morbidity and mortality from infection [73, 74]. In multiple sclerosis, thymic rebound after autologous HSCT was associated with a favorable clinical response [75]. In heart transplant recipients, higher TREC levels during rejection have been described in a small pilot study [76]. Considering how suboptimal TREC assays are in measuring thymic output in adults, these promising results indicate that accurate measurement of thymic output could be useful in predicting clinically significant outcomes and guide personalized medicine.

More recently, the efficacy of immune-oncological treatments, such as PD-1 checkpoint inhibitor therapy, has been shown to critically depend on the patient’s immune composition prior to receiving the treatment [77]. Patients that respond well have higher baseline T cell proliferation and a greater presence of antigen-experienced stem-cell-like T cells compared to exhausted T cells [77]. Moreover, checkpoint inhibitors appear to be less effective in older patients, likely due to age-related changes of the immune system, such as altered thymic output [78]. Interestingly, higher proportion of naïve CD8 cells appeared to predict response to a combination check-point inhibitor treatment in hepatocellular carcinoma [79].

## Development of new methods to measure thymic output

TREC assays as a means to quantify thymic output in HIV infection were first described by Douek et al. in 1998 [43, 44]. Yet, despite the known limitations of the TREC assays, no alternative method currently exists for reliably measuring CD8 + RTEs. Given the impracticality and invasiveness of accessing thymus tissue, thymic output should ideally be measurable through blood samples. Achieving this requires improved cellular RTE markers for flow cytometry, a method widely used in clinical laboratories. The recent large-scale adoption of single-cell RNA sequencing may provide candidate markers [67, 6], but these would require thorough validated before being established for clinical use. An ideal RTE marker for clinical application should fulfill the following criteria: Its expression should be sufficiently high to enable reliable detection using standard antibodies and flow cytometers available in clinical laboratories; it should correlate strongly with TREC levels; optimally, its expression should demonstrate a clear relationship with altered thymic output in clinical contexts, such as after thymectomy or during T cell reconstitution following hematopoietic stem cell transplantation; and finally, its kinetics must align with the physiological decline of thymic function with age.

## Concluding remarks

RTEs serve as a direct link between the thymus and the periphery. Thymic involution is a key factor contributing to the decline of immune function with age. Therefore, accurately measuring thymic output and identifying RTEs is essential for studying, and potentially mitigating immune system ageing, as well diagnosing and monitoring immune deficiencies, and understanding T cell biology in the context of infection. To measure thymic output more precisely and improve our understanding of the aging immune system’s impact in various clinical settings, new innovations are urgently needed. Moreover, in addition to new methods, better universal standardization and quality assurance should be implemented to measuring of thymic output so that results produced by different clinical laboratories would be comparable [80, 81].
